# Enrofloxacin Rapid Detection in Aquatic Foods: Based on DNA Aptamer Sensor

**DOI:** 10.3390/foods13060941

**Published:** 2024-03-20

**Authors:** Xiuli Bao, Siyuan Wang, Qingfang Hao, Yue Bai, Siying Li, Shuai Zhang, Lei Zhang, Xinxin Kang, Mingsheng Lyu, Shujun Wang

**Affiliations:** 1 Jiangsu Key Laboratory of Marine Bioresources and Environment/Jiangsu Key Laboratory of Marine Biotechnology, Jiangsu Ocean University, Lianyungang 222005, China; xlbao@jou.edu.cn (X.B.); siyuanwang@jou.edu.cn (S.W.); qfhao@jou.edu.cn (Q.H.); yuebai@jou.edu.cn (Y.B.); 2022220837@jou.edu.cn (S.L.); 2022210128@jou.edu.cn (S.Z.); leizhang@jou.edu.cn (L.Z.); kangxinxin@jou.edu.cn (X.K.); mslyu@jou.edu.cn (M.L.); 2 Co-Innovation Center of Jiangsu Marine Bio-Industry Technology, Jiangsu Ocean University, Lianyungang 222005, China

**Keywords:** enrofloxacin, aptamer, SELEX, antibiotic residues, aquatic products

## Abstract

Enrofloxacin (ENR) is widely used as a synthetic fluoroquinolone antibiotic for disease control in aquatic animals. ENR aptamers were screened in this study using the magnetic bead-SELEX method, and a graphene oxide fluorescent sensor was developed to detect the ENR residues in aquatic products. Firstly, ENR was conjugated to amino magnetic beads by amidation reaction, and then the aptamer sequences showing high affinity to ENR were screened step by step by using the SELEX screening method. Finally, after 10 rounds of SELEX screening, six candidate aptamers with high affinity were obtained. Among these, ENR-Apt 6 was selected based on its secondary structure features, high affinity (*K_d_* = 35.08 nM), and high specificity to ENR. Furthermore, a fluorescent sensor was prepared using graphene oxide and ENR-Apt 6. The results showed that the linear range of the sensor could reach 600 nM (R^2^ = 0.986), while its optimal linear range was 1–400 nM (R^2^ = 0.991), with the lowest detection limit of 14.72 nM. The prepared sensor was successfully used for the detection of ENR in real samples, with a recovery range of 83.676–114.992% and a relative standard deviation < 10% for most of the samples.

## 1. Introduction

With rich aquatic resources, China is the world’s leading country in terms of production of aquatic products and aquaculture [1,2]. However, the expanding scale of aquaculture has led to the accumulation of residual bait and excreta in the water, which has caused various types of diseases and raised grave concerns [3,4,5]. Currently, antibiotics are commonly used to prevent and treat animal infectious diseases [6]. Enrofloxacin (ENR), a synthetic fluoroquinolone antibiotic, is classified as an animal-specific drug and is widely used to control diseases in aquatic organisms [7,8]. However, the widespread use of antibiotics has led to the problem of bacterial resistance [9,10], which caused aquatic animals to exhibit symptoms such as immune system damage, physiological abnormalities, and gut flora dysbiosis [11,12]. Furthermore, human health and environmental ecology would be affected along with the enrichment effect in organisms [13,14,15].

High-performance liquid chromatography (HPLC) or HPLC-mass spectrometry (HPLC-MS) [16], thin-layer chromatography surface-enhanced Raman spectroscopy (TLC-SERS) [17,18], enzyme-linked immunosorbent assay (ELISA) [19], immunochromatographic assay [20], and other methods have been employed in the majority of prior studies to determine ENR. While these traditional detection techniques are sensitive and precise, their instrumentation and sample preparation processes are expensive, time-consuming, and complicated [21]. Aptamer-based biosensors are currently progressively becoming the best substitute for conventional techniques in the detection of antibiotic residues [22]. 

Aptamer is a single-stranded RNA or DNA fragment that can be selected by systematic evolution of exponentially enriched ligands (SELEX) [23,24,25]. It can fold into different three-dimensional structures upon binding to target molecules, thus achieving specific recognition of target molecules such as proteins [26], metal ions [27], bacteria [28,29], and antibiotics [5]. As for SELEX, it mainly includes magnetic bead-SELEX (MB-SELEX) [30,31], graphene oxide-SELEX (GO-SELEX) [32], Cell-SELEX [33], and capillary electrophoresis-SELEX (CE-SELEX) [34]. Dolati S et al. [35] selected ENR aptamers by using SELEX and synthesized a fluorescent molecular probe to achieve specific detection of ENR with a limit of detection of 3.7 nM. Wen X et al. [36] screened and identified nitrofurazone aptamers based on MB-SELEX with a linear range of 1.25~160 ng/mL and a limit of detection of 1.13 ng/mL. In addition, Guan J et al. [37] selected a highly specific aptamer for penicillin G by the GO-SELEX method, and the limit of detection reached 0.05 nM. The sensors based on aptamers have shown great potential for application in real samples.

In this study, the MB-SELEX method was used to screen the corresponding aptamers for ENR, and a fluorescent sensor was prepared for the detection of ENR using graphene oxide (GO). The sensor achieved rapid detection of ENR in aquatic foods on site. The sensor has the characteristics of high specificity, low cost, easy to store, and simple operation. Our results provided a new method for the control of harmful substances in aquatic products.

## 2. Materials and Methods

### 2.1. Chemicals and Materials

ENR, ofloxacin (OFL), and ciprofloxacin (CIP) were purchased from Yuanye Biological Co., Ltd. (Shanghai, China), while sulfadiazine (SD), roxithromycin (RXM), tobramycin (TOB), and clarithromycin (CLT) were acquired from Sangon Biotech Co., Ltd. (Shanghai, China) Amino magnetic beads and chemicals including GO colloidal solution of analytical grade, N-2-hydroxyethylpiperazine-N′-2-ethanesulfonic acid (HEPES), anhydrous magnesium chloride, ethylenediaminetetraacetic acid, N,N-diisopropylethylamine (DIPEA), 1-hydroxybenzotriazole monohydrate (Hobt), and tris-pyrrolidinyl phosphonium bromide hexafluorophosphate (Pybrop) were procured from Aladdin (Shanghai, China).

Random oligonucleotide sequences: forward primer (GCGGCTGCTTACGGATAGAA); reverse primer (GACTTGGGCTTTACGGATGG); and Library (GCGGCTGCTTACGGATAGAA-N_35_-CCATCCGTAAAGCCCAAGTC) were prepared at Sangon Biotech Co., Ltd. (Shanghai, China)

### 2.2. Methods

#### 2.2.1. Immobilization of ENR

**Figure 1:**
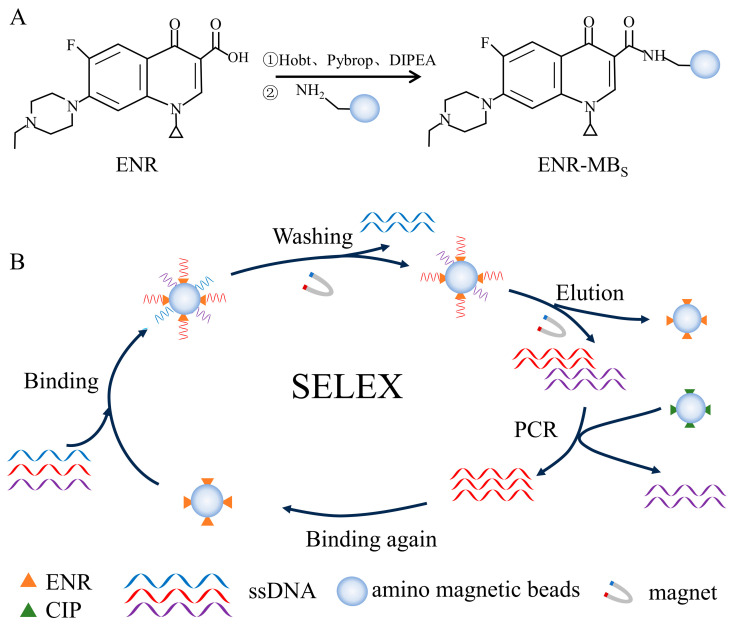
Schematic diagram of ENR aptamer screening: (**A**) ENR immobilization, (**B**) SELEX-based in vitro screening of aptamers.

Immobilization of ENR was carried out using an amidation reaction. As shown in Figure 1A, ENR was coupled with amino magnetic beads using Hobt, Pybrop, and DIPEA as carboxyl activators. Firstly, 5 mM ENR (200 μL) was mixed with 65 mM Hobt (100 μL), 20 mM Pybrop (200 μL), and 20% DIPEA (100 μL). The mixture was incubated at 25 °C for 2 h in a thermostatic mixer. Afterward, the activated ENR solution was stored at 4 °C in a refrigerator. Then, 100 μL of amino magnetic beads were taken in a centrifuge tube (1.5 mL) and magnetically separated. Subsequently, 100 μL of ultrapure water and 100 μL of methanol were added in turn and washed the magnetic beads three times. After magnetic separation, the magnetic beads were added to 150 μL of activated ENR solution and incubated overnight at 25 °C to obtain ENR encapsulated by amino magnetic beads. As shown in Figure S1, the same process was followed for CIP coupling.

In summary, the magnetic beads incubated overnight were magnetically separated, and then the fluorescence intensity of the antibiotic solution was measured using an INFINIITE M1000 PRO multifunctional enzyme marker at an excitation wavelength of 280 nm and emission wavelength of 350–550 nm to determine the effect of ENR and CIP immobilization.

#### 2.2.2. In Vitro Screening of Aptamers

A total of 10 rounds of in vitro screening were performed using the SELEX method. Each round of screening was negatively screened using unloaded magnetic beads. The incubation time was reduced in the 5th round of screening to increase sensitivity, followed by the addition of CIP-coated magnetic beads as a negative screen in the 8th round of screening. Reducing the incubation time could enhance the selection of higher affinity aptamers. Similarly, a negative screen could remove the sequences that could specifically bind CIP. The entire screening process is shown in Figure 1B. The random ssDNA library was heated at 95 °C for 5 min and subsequently placed on ice for 15 min, after which it was gradually returned to room temperature. Before the screening, the unloaded magnetic beads were washed three times with 100 μL of selection buffer (100 mM HEPES, 300 mM NaOH, 30 mM MgCl_2_, and 0.02% Tween 20, pH 7.5). After washing, the magnetic beads were magnetically separated and incubated with the random libraries at 25 °C for 1 h. Then, the supernatant was recovered and added to the ENR-coated magnetic beads and incubated for 2 h at 25 °C. After magnetic separation, the magnetic beads were washed three times with selection buffer, and then added 100 μL of elution buffer (50 mM Tris-HCl, 10 mM EDTA-2Na-2H_2_O, 3 M Urea, and 0.02% Tween 20, pH 8.0) and incubated at 95 °C for 5 min. The elution processing repeated three times. The eluent was collected by alcoholic precipitation. Finally, the concentration of ssDNA was measured using a UV–visible spectrophotometer to calculate the recovery of aptamer. 

#### 2.2.3. Sequencing and Structural Analysis

After 10 rounds of screening, PCR products obtained in the last round were sequenced at Sangon Biotech Co., Ltd. (Shanghai, China). The homology of candidate sequences was analyzed by MAGE 11 (Molecular Biology and Evolution, Tokyo, Japan). Secondary structures of DNA samples were predicted using DNA Fold Webserver (DNA Folding Form (unafold.org). Based on the homology, secondary structures, and the minimum Gibbs free energies of the candidate aptamers, six aptamer sequences with higher enrichment rates were finally selected. Subsequently, 6-carboxyfluorescein (6-FAM) tags were added to the 5′ ends of the selected aptamers for subsequent experiments.

#### 2.2.4. Affinity of Aptamers to ENR 

The dissociation constant (*K_d_*) of the candidate aptamer indicates the affinity of the aptamer to the target molecule, with a lower *K_d_* representing higher affinity. GO is a hexagonally arranged, two-dimensional (2D) crystal structure composed of carbon, with various oxygen-containing functional groups, such as carboxyl, hydroxyl, and epoxy groups, present on its surface. GO can quench the FAM by changing the fluorescent signals through its conjugated structure [38,39].

Consequently, the *K_d_* of each selected aptamer was determined using the GO fluorescence quenching method. First, the aptamers were dissolved in ultrapure water at concentration gradients of 25, 50, 75, 100, 125, 150, 175, and 200 nM to determine their ENR affinities. Then, 0.2 μg/mL, 0.4 μg/mL, 0.6 μg/mL, 0.8 μg/mL, 1 μg/mL, 10 μg/mL, 20 μg/mL, and 30 μg/mL of GO solutions were prepared to determine the optimal concentration of GO to quench the fluorescence of aptamers. After determining the optimal concentration, 100 μL of aptamer solution was mixed with 100 μL of optimal GO solution (20 μg/mL), followed by the addition of 100 μL of 250 μM ENR solution. A volume of 100 μL of ultrapure water was used as the control. Finally, the fluorescence intensity of the solution was measured by INFINIITE M1000 PRO multifunctional enzyme marker at an excitation wavelength of 492 nm and an emission wavelength of 518 nm. Non-linear fitting analysis was performed using Origin 2018 software, and *K_d_* values of the aptamers were calculated by using the following non-linear regression equation:

(1) $$Y=B_{max}\times X÷\left(K_{d}+X\right)$$

Here, *Y* represents the fluorescence intensity of the supernatant, *B_max_* represents the number of maximum binding sites, and *X* represents the concentration of the aptamer.

#### 2.2.5. Development of ENR Aptamer-Based Sensor

ENR aptamer-based sensors were created using the aptamers labeled with FAM based on GO fluorescence quenching. GO adsorbs the aptamer using the hydrophobic groups and the “π-π” stacking interactions between ssDNA and GO. Furthermore, GO quenches the fluorescence signals of the modified FAM tagged to the 5′ end of the aptamer. Because trehalose is widely used in the food industry due to its high safety and stability [40], GO–aptamer complexes were dried with trehalose to prepare a simple and convenient sensor.

Both GO concentration and aptamer concentration play a vital role in the performance of the sensor. Too low of a concentration of GO leads to incomplete quenching of aptamer, while too high of a GO concentration leads to ineffective fluorescence recovery after ENR addition. Therefore, different concentrations of GO (1 μg/mL, 20 μg/mL, 30 μg/mL, 40 μg/mL, and 50 μg/mL) were used. Similarly, different aptamer concentrations (200 nM, 400 nM, 600 nM, and 800 nM) were used to determine the optimal aptamer concentration for the performance of the sensor. Firstly, the aptamer and GO (ratio 1/1, *v*/*v*) were added into a centrifuge tube (0.2 mL), and the fluorescence quenching effect was observed. Then, 10 μL of 1 μM aptamer, 10 μL of GO solution, and 10 μL of trehalose were taken into a centrifuge tube and mixed. This mixed solution was dried for 8 h, and then 1 μM of ENR was added to the solution to observe the fluorescence recovery effect. The fluorescence intensities of solutions were measured by an INFINIITE M1000 PRO multifunctional enzyme marker at an excitation wavelength of 492 nm and emission wavelength of 518 nm, and photographs were taken using a blue light illuminator.

Finally, to develop the sensor, a 0.2 mL centrifuge tube was used as the sensor carrier, containing 10 μL of 0.25 M trehalose, 10 μL of aptamer (optimal concentration: 600 nM), and 10 μL of GO (optimal concentration: 20 μg/mL). After homogenous mixing of components, the centrifuge tube was placed in a thermostatic mixing apparatus at 50 °C for 8 h. Then, the sensor was dried for 8 h at 50 °C.

#### 2.2.6. Characterization of ENR Aptamer-Based Sensor

Specificity, sensitivity, and stability are crucial indicators of the performance of the sensor. To evaluate the specificity of the selected aptamers, OFL, CIP, SD, RXM, TOB, and CLT antibiotics were used as the structural analogs of ENR. Firstly, 1 mM ENR and its structural analogs were added to the already-dried sensors. After mixing well, 100 μL of antibiotic solutions were pipetted out and transferred to an enzyme-labeled plate. The fluorescence intensities of solutions were measured at an excitation wavelength of 492 nm and an emission wavelength of 518 nm.

Ultrapure water was added to the dried sensor as the control to examine the sensitivity of the sensor. Simultaneously, ENR solutions of 1, 25, 50, 100, 150, 200, 250, 300, 350, 400, 500, 600, 800, and 1000 nM concentrations were added to the sensors and mixed thoroughly. Then, the fluorescence intensities of solutions were measured and photographed by using a blue light illuminator.

Next, sensor stability was investigated by leaving the dried sensors at room temperature for 1, 3, 7, 14, 21, 30, 45, and 60 d. After completion of the designated time period, 1 μM ENR solution was added to the sensor and mixed thoroughly, and then the fluorescence intensity of the solution was measured and photographed using a blue light illuminator.

Finally, the sensitivity of the sensor was checked for detection of ENR in real samples. The muscular tissues of *Parabramis pekinensis* (1 g) were ground with 10 mL of aqueous acetonitrile solution (80:20, *v*:*v*). Then, the ground muscular tissues were homogenized and centrifuged at 8000× *g* for 10 min, and the extracts were collected and filtered through a 0.22 µm membrane to remove other impurities. The tissue extracts were mixed with different concentrations of ENR, including 5, 50, 100, 150, 200, 300, 400, 600, 800 and 1000 nM, and then the fluorescence intensities of solutions were measured and photographed on a blue light illuminator.

#### 2.2.7. Analysis of the Real Aquatic Samples

Real samples of aquatic products were tested to detect the ENR residues. *Larimichthys polyactis, Portunus trituberculatus*, *Lateolabrax japonicus*, and *P. pekinensis* were purchased from the Lianyungang Supermarket, and the tissue samples were processed according to the method described in Section 2.2.6. The obtained tissue extracts were then spiked with ENR of different concentrations (i.e., 15 nM, 28 nM, and 50 nM). Each set of experiments was repeated three times, and the spiked ENR recovery and relative standard deviation (RSD) were calculated.

#### 2.2.8. Data Analysis and Statistics

Three sets of parallel experiments were set up for experiments. Figures were made using Origin 2018.

## 3. Results and Discussion

### 3.1. Immobilization of ENR

**Figure 2:**
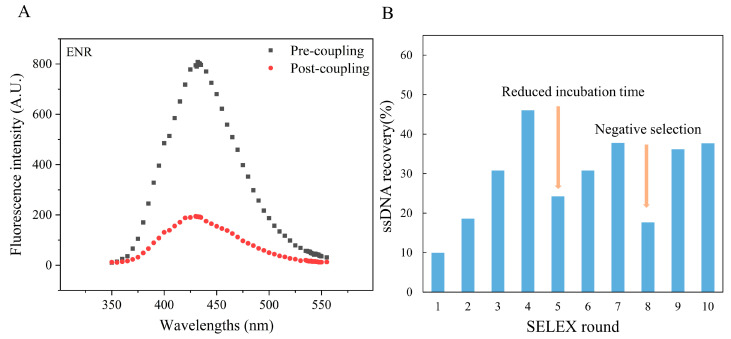
Characterization of ENR aptamers: (**A**) Changes in the fluorescence intensity of ENR solution before and after coupling; (**B**) ssDNA recovery rate during each round of aptamer screening by SELEX.

In this experiment, the ENR coupling rate was found to be stable in the range of 56–85%. Due to the formation of the “π-π” conjugation system, the ENR molecule has the ability to self-fluorescence. Therefore, the change in the fluorescence intensity of the ENR solution before and after coupling was measured to confirm the successful immobilization of ENR on the amino magnetic beads. As shown in Figure 2A, the decrease in fluorescence intensity in the supernatant after coupling proved that most of the ENRs were immobilized on the amino magnetic beads, and the coupling rate was around 76% after the 7th round of coupling. Moreover, CIP was also immobilized on amino magnetic beads with a coupling rate of approximately 63% (Figure S2). Previously, other antibiotics have also been successfully immobilized on magnetic beads using this principle. For example, Wen X et al. [36] reported a coupling rate of 71.8% for nitrofurazone (NFZ) aptamer. Similarly, Ni H et al. [41] observed a coupling rate of 80% during immobilization of ENR on magnetic beads.

### 3.2. In Vitro Screening 

As shown in Figure 2B, the ssDNA recovery rate gradually increased as the screening process proceeded. However, a decrease in recovery rate was observed in the 5th and 8th rounds of screening, which may be due to the decreased incubation time and the addition of CIP-coated magnetic beads. This led to increased screening pressure, which caused the elution of the low-binding ssDNA sequences. Overall, the ssDNA recovery rate showed an increasing trend, with a relatively stable recovery rate in the 10th round of screening. Therefore, the screening products of the 10th round were sent to Sangon Biotech Co., Ltd. (Shanghai, China) for sequencing.

During the in vitro screening, the ssDNA recovery rate may reflect the enrichment of high-affinity sequences to some extent. For example, when Wen X et al. [36] screened the NFZ aptamers, the ssDNA recovery rate increased round by round and eventually reached more than 70%. Similarly, when Shi M et al. [5] screened the FFA aptamers, the ssDNA recovery rate was found to be stable at around 55%. Compared with the other studies, the recovery rate of ssDNA in this experiment was not very high and was stable at around 35% only. The low recovery rate of ssDNA may be attributed to the addition of unloaded amino magnetic beads in each round for reverse screening. Because the amino beads have abundant binding sites, several non-specific binding sequences might have been removed by the beads, resulting in a lower ssDNA recovery rate. However, interestingly the sequencing results revealed the enrichment of sequences with ENR-specific binding sites, with the highest enrichment rate of 21.6% (Table S1). These findings suggest the high enrichment rate of MB-SELEX method.

### 3.3. Sequencing and Structural Analysis of Aptamers

**Figure 3:**
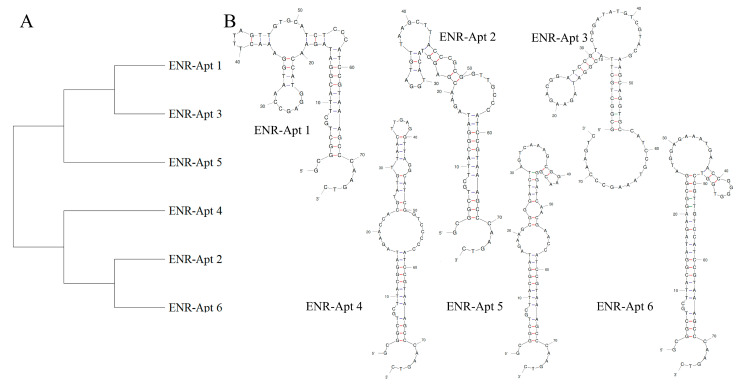
Analysis of aptamer sequences: (**A**) aptamer homology analysis by MAGE 11; (**B**) prediction of the secondary structures of aptamers by unafold.org.

During the 10 rounds of in vitro screening, a total of 112,755 aptamer sequences were screened. Among these, the six aptamer sequences accounting for the enrichment ratio were selected for further experiments (Table S1). Homology analysis of the six sequences by MEGA 11 revealed that these aptamers belong to two families. As shown in Figure 3A, ENR-Apt 1, ENR-Apt 3, and ENR-Apt 5 belong to one family, while ENR-Apt 2, ENR-Apt 4, and ENR-Apt 6 belong to the other family. Next, the complete secondary structures of the six candidate sequences were simulated using DNA Fold Webserver, as shown in Figure 3B. The six candidate sequences were observed to have 1–3 stem-loop structures as well as GC-rich sequences such as “CCGAGG”, “GGA”, “ACGG”, “ CGGGGG”, etc. The GC base pairing increases the stability of the stem-loop structure, thus enhancing the affinity of aptamers with the target [42]. This finding indicates the high affinity of aptamers to ENR. 

It is now widely accepted that the recognition mechanism between nucleic acid aptamers and target molecules is either mediated through the formation of specific structures, such as stem-loop, hairpin, G-tetrahedron, etc., or by bonding between aptamers and targets through intermolecular hydrogen bonding, electrostatic interactions, and other forces [43]. Gao J et al. [44] truncated two stem-loop structures in the original aptamer sequence and confirmed that the truncated stem-loop structure caused stronger recognition of α-amanitin by the aptamer. Similarly, Li Y et al. [42] found that the secondary structure of the screened aptamer APTZO-1 contained a single pocket structure with multiple stem-loop structures.

### 3.4. Affinity of Aptamer

GO concentration is a key factor affecting the detection performance of aptamer-based sensors. As shown in Figure S3, the fluorescent groups were not completely quenched when the GO concentration was low. On the other hand, complete quenching of fluorescent groups was observed at a GO concentration of 20 μg/mL. At higher concentrations of GO, fluorescent signals could not be effectively recovered after the addition of ENR solution. Therefore, 20 μg/mL was selected as the optimal concentration of GO for the subsequent experiments.

**Figure 4:**
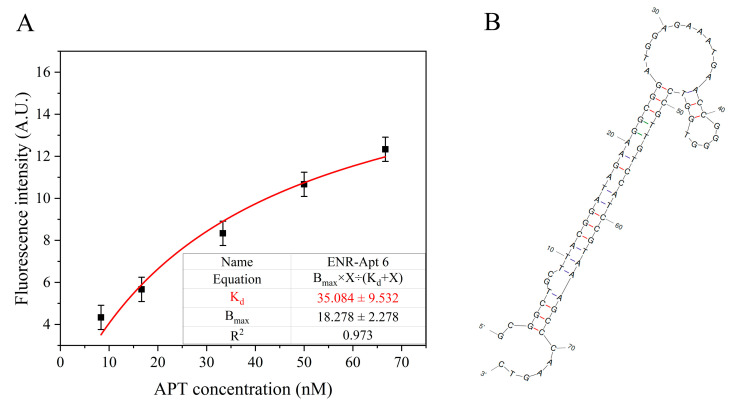
ENR-Apt 6 affinity analysis: (**A**) Changes in fluorescence intensity with increased concentration of ENR-Apt 6; (**B**) secondary structure of ENR-Apt 6 (predicted by unafold.org).

As shown in Figure S4, the *K_d_* values of the six aptamers are less than 50 nM, which indicate the successful screening of aptamer sequences with high affinity. Figure 4A shows the non-linear fitting curve of ENR-Apt 6, with a *K_d_* value of 35.08 nM. Figure 4B shows the predicted secondary structure of ENR-Apt 6, containing a stem-loop structure. In addition, ENR-Apt 6 has the lowest minimum Gibbs free energies (dG) and higher GC content (Table S1), which indicates that the structure of this candidate aptamer is the most stable among the six selected aptamers. Because ENR-Apt 6 showed good structural characteristics and high binding affinity for ENR, it was used for subsequent ENR assays.

### 3.5. Development of ENR Aptamer-Based Sensor

**Figure 5:**
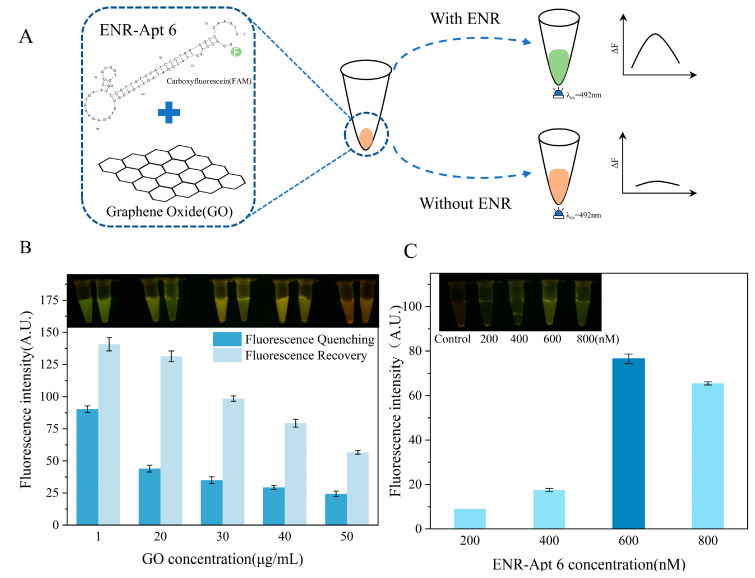
Development of ENR aptamer-based sensor and optimization of GO and aptamer concentrations: (**A**) schematic diagram showing the principle of ENR aptamer-based sensor; (**B**) optimization of GO concentration; (**C**) optimization of the concentration of ENR-Apt 6.

As shown in Figure 5A, the GO–aptamer complex was immobilized at the bottom of the centrifuge tube. In the presence of an ENR, ENR-Apt 6 was competitively displaced from the GO, leading to the formation of an ENR–aptamer complex in the solution. Furthermore, the fluorescence signal of the FAM label was restored. On the contrary, no aptamer ENR-Apt 6 displacement occurred in the absence of ENR, showing no fluorescence signal.

As shown in Figure 5B, the fluorescence quenching effect of GO is not significant at a concentration of 1 μg/mL, and there is a strong interference by the background fluorescence. With the increase in concentration, the ability of GO to quench the aptamer fluorescence enhanced, but the fluorescence intensity did not change significantly. Furthermore, the addition of ENR solution led to competitive displacement of ENR-Apt 6 from the GO–aptamer complex, resulting in an increase in the fluorescence signal. However, the fluorescence recovery gradually decreased with the increase in GO concentration. A comprehensive comparison concluded that better fluorescence quenching and fluorescence recovery effects were observed at a GO concentration of 20 μg/mL. In addition, the highest fluorescence intensity was observed when the initial concentration of ENR-Apt 6 added to the sensor was 600 nM (Figure 5C).

### 3.6. Characteristics of ENR Aptamer-Based Sensor

**Figure 6:**
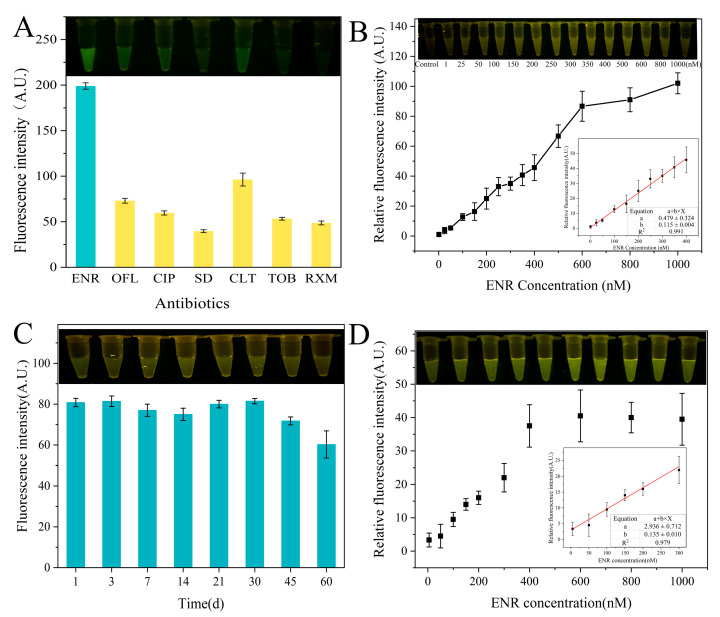
Performance of ENR aptamer-based sensor: (**A**) specificity of ENR-Apt 6 for ENR compared to other antibiotics (ofloxacin (OFL), ciprofloxacin (CIP), sulfadiazine (SD), clarithromycin (CLT), tobramycin (TOB), and roxithromycin (RXM)); (**B**) sensitivity of sensors at different concentrations of ENR; (**C**) stability of sensors after different storage periods (1, 3, 7, 14, 21, 30, 45, 60 d); and (**D**) sensitivity of sensors at different concentrations of ENR in *P. pekinensis* samples.

As shown in Figure 6A, the fluorescence intensities in the presence of ENR are higher than those in the presence of other antibiotics. This indicates the specificity of a developed sensor for ENR detection. In addition, the linear range of the GO fluorescent aptamer sensor reached up to 600 nM (R^2^ = 0.986), while its optimal linear range was 1–400 nM (R^2^ = 0.991), with a low detection limit of 14.72 nM for ENR (Figure 6B). A number of ENR detection methods have been established in previous studies (Table 1). Compared with the traditional analytical methods (HPLC and SERS), our sensor did not require precise instruments. Also, the operation was easy, and detection could be performed rapidly on-site. Moreover, our sensor has a wider linear range and lower LOD. 

Furthermore, Figure 6C shows the stability of the sensor after storage at room temperature. The fluorescence intensity was relatively stable when the sensor was stored at room temperature for less than 30 d, while it slightly decreased after storage for 45 d and 60 d. This indicates the good stability of the sensor for 30 d. Overall, the developed sensor shows a wider linear range, a low detection limit, a long storage time, and a good application prospect for ENR detection. Although the presence of certain background fluorescence signals in the real samples decreased the relative fluorescence intensity, the linear range of fluorescence detection by the sensor in the *P. pekinensis* sample could reach 300 nM (Figure 6D).

### 3.7. Analysis of Real Samples

To further validate the feasibility of ENR detection by ENR-Apt 6-GO sensor, recovery experiments were performed, and the results are shown in Table 2. The extracts of *L. polyactis*, *P. trituberculatus*, *L. japonicus*, and *P. pekinensis* were spiked with ENR at three different concentrations (15 nM, 28 nM and 50 nM). The results show that the ENR recoveries are in the range of 83.676–114.992%, with RSD < 10% for most of the samples. These findings prove the potential of this sensor to detect ENR in real aquatic samples. The recoveries of a few samples are over 100% (Table 2), and it suggests some samples contained components that could affect the fluorescence. 

Aptamer-based biosensors for the detection of antibiotic residues in animal foods have developed rapidly [43]. The design of the original library and the selection of the screening technique are all crucial to the whole selecting process. In this study, ENR immobilization on magnetic beads, the negative screening, specifically empty magnetic beads for negative screening, could enhance the target aptamers enrichment ratio [5,36,37,48]. Compared with large-scale equipment, portable and simple detection components could meet the requirements of on-site detection [22]. In this study, a fluorescent biosensor based on the quenching of GO was designed. The sensor has a low LOD and a wide linear range. Meanwhile, GO has good properties, such as water solubility and biocompatibility [35]. So, the sensor could be used on-site and was disposable. However, the real samples contain rich proteins, lipids, etc., and the composition is more complicated. Eliminating interference in biological samples remains a challenge in the application of nucleic acid aptamer sensors. 

## 4. Conclusions

In this study, ENR aptamers were immobilized on amino magnetic beads by amidation reaction, and then in vitro screening of aptamers was performed by the SELEX method. After 10 rounds of SELEX screening, six aptamers with high enrichment rates were obtained. After a comprehensive comparison of secondary structures and binding affinities, ENR-Apt 6 (*K_d_* value of 35.08 nM) was finally selected for the development of the sensor. Subsequently, a fluorescent biosensor was prepared using GO and ENR-Apt 6. Furthermore, the developed sensor showed a lower limit of detection of 14.72 nM and an optimal linear range of 400 nM, with high specificity and stability. This sensor was successfully applied for the detection of ENR in real aquatic samples.

## Figures and Tables

**Table 1:** Comparison of enrofloxacin detection with reported methods.

Method	Linear Range	LOD	Reference
HPLC-MS	--	0.1 µg/kg	[16]
TLC-SERS	--	12.6 ng/mL	[17]
Graphene oxide-based label-free fluorescent assay	5–250 nM	3.7 nM	[35]
DNA tweezers fluorescence aptasensor	0.01–100 ng/mL	0.008 ng/mL	[45]
Based on G-quadruplex structure-switching aptamer	0.05–20 µM	26.7 nM	[46]
terbium (III) and aptamer-based probe	1.0–100 ng/mL	0.061 ng/mL	[47]
This work	1–600 nM	14.72 nM	

**Table 2:** Recovery of ENR in real samples.

Sample	Spiked ENR Concentration (nM)	Measured Concentration (nM)	Recovery (%)	RSD (%, *n* = 3)
*Larimichthys polyactis*	15	16.145	107.634	12.444
	28	24.340	87.555	6.839
	50	57.496	114.992	9.613
*Portunus trituberculatus*	15	17.610	117.398	14.566
	28	23.492	83.676	1.757
	50	51.905	103.811	11.219
*Lateolabrax japonicus*	15	16.044	106.958	6.414
	28	29.480	105.287	2.418
	50	56.628	113.256	1.614
*Parabramis pekinensis*	15	13.469	91.114	4.347
	28	23.489	84.494	5.419
	50	47.655	95.310	1.392

## Data Availability

The original contributions presented in the study are included in the article/Supplementary Material, further inquiries can be directed to the corresponding author.

## Supplementary Materials

The following supporting information can be downloaded at: https://www.mdpi.com/article/10.3390/foods13060941/s1, Figure S1: Schematic diagram of CIP immobilization; Figure S2: CIP fluorescence intensity in solution before and after coupling; Figure S3: Effect of fluorescence intensity on GO concentration; Figure S4: Six candidate adaptors non-linear fitting curves; Table S1: Candidate aptamer sequences and their characteristics.
