# Supplementary material for: Enrofloxacin Rapid Detection in Aquatic Foods: Based on DNA Aptamer Sensor
Source: Foods. 2024 Mar 20;13(6):941. doi: 10.3390/foods13060941 (PMC10969504; doi:10.3390/foods13060941)
Supplement: Supplementary file 1 [file foods-13-00941-s001.zip › foods-2891866-supplementary.pdf]

Enrofloxacin Rapid Detection in Aquatic Foods: based on DNA Aptamer Sensor

Xiuli Bao[1,2], Siyuan Wang[1,2], Qingfang Hao[1,2], Yue Bai[1,2], Siying Li[1,2], Shuai Zhang[1,2], Lei Zhang[1,2], Xinxin Kang[1,2], Mingsheng Lyu [1,2], Shujun Wang[1,2,*]

[1]Jiangsu Key Laboratory of Marine Bioresources and Environment/Jiangsu Key Laboratory of Marine Biotechnology, Jiangsu Ocean University, Lianyungang, 222005, China
[2]Co-Innovation Center of Jiangsu Marine Bio-industry Technology, Jiangsu Ocean University, Lianyungang, 222005, China
***Correspondence:** sjwang@jou.edu.cn

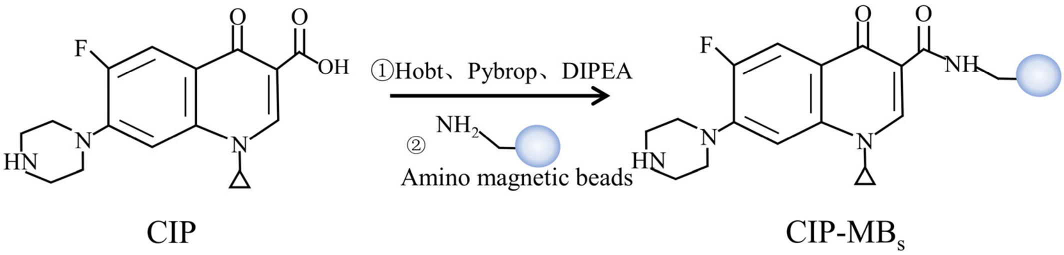

Figure.S1. Schematic diagram of CIP immobilization

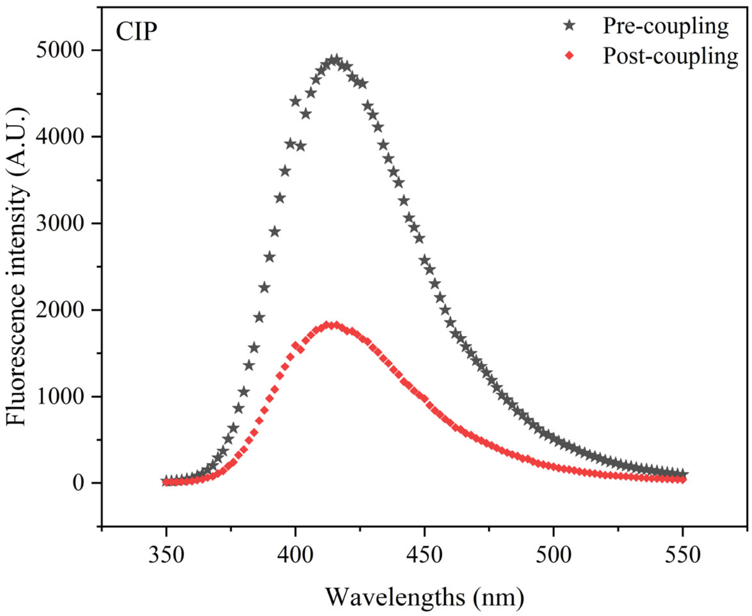

Figure.S2. CIP fluorescence intensity in solution before and after coupling

Table.S1 Candidate aptamer sequences and their characteristics

Name	Sequence（5’-3’）	enrichment ratio （%）	dG （kcal/mol）	GC （%）
ENR-Apt 1	GCGGCTGCTTACGGATAGAACCATGG AGCCAATGGAAACTTTAGTTGTGCATC TCCCATCCGTAAAGCCCAAGTC	21.2	-8.29	56
ENR-Apt 2	GCGGCTGCTTACGGATAGAACGAGGT ACATGGATGTTAAGCTTACCCGCGGTT GCCCATCCGTAAAGCCCAAGTC	8.62	-8.84	60
ENR-Apt 3	GCGGCTGCTTACGGATAGAAGACGGA TCCGTCGGATATGTCGTACGAAGCAGG TGCCATCCGTAAAGCCCAAGTC	2.74	-10.92	60
ENR-Apt 4	GCGGCTGCTTACGGATAGAACCACGT ATGTTAACTTGAGGTTAGGCATCGGTC CCCCATCCGTAAAGCCCAAGTC	2.47	-8.14	60
ENR-Apt 5	GCGGCTGCTTACGGATAGAAGCGGGA TCTAGTCAAAGCGGAACGGGATCAAC GAACCATCCGTAAAGCCCAAGTC	1.44	-10.01	64
ENR-Apt 6	GCGGCTGCTTACGGATAGAAGGCGGA TGGAGAAATGAACCGGGGTGGTCCGT TGTCCATCCGTAAAGCCCAAGTC	0.69	-11.15	64

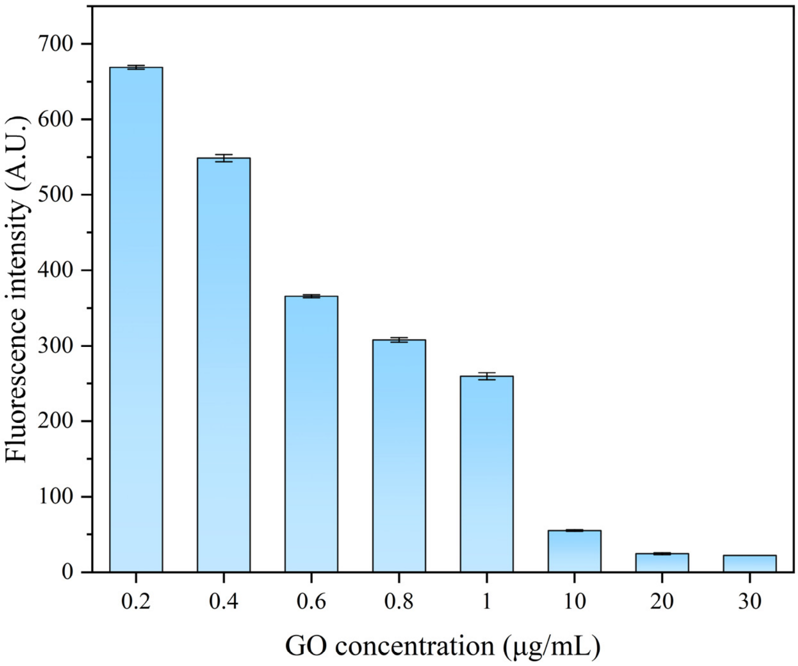

Figure.S3. Effect of fluorescence intensity on GO concentration

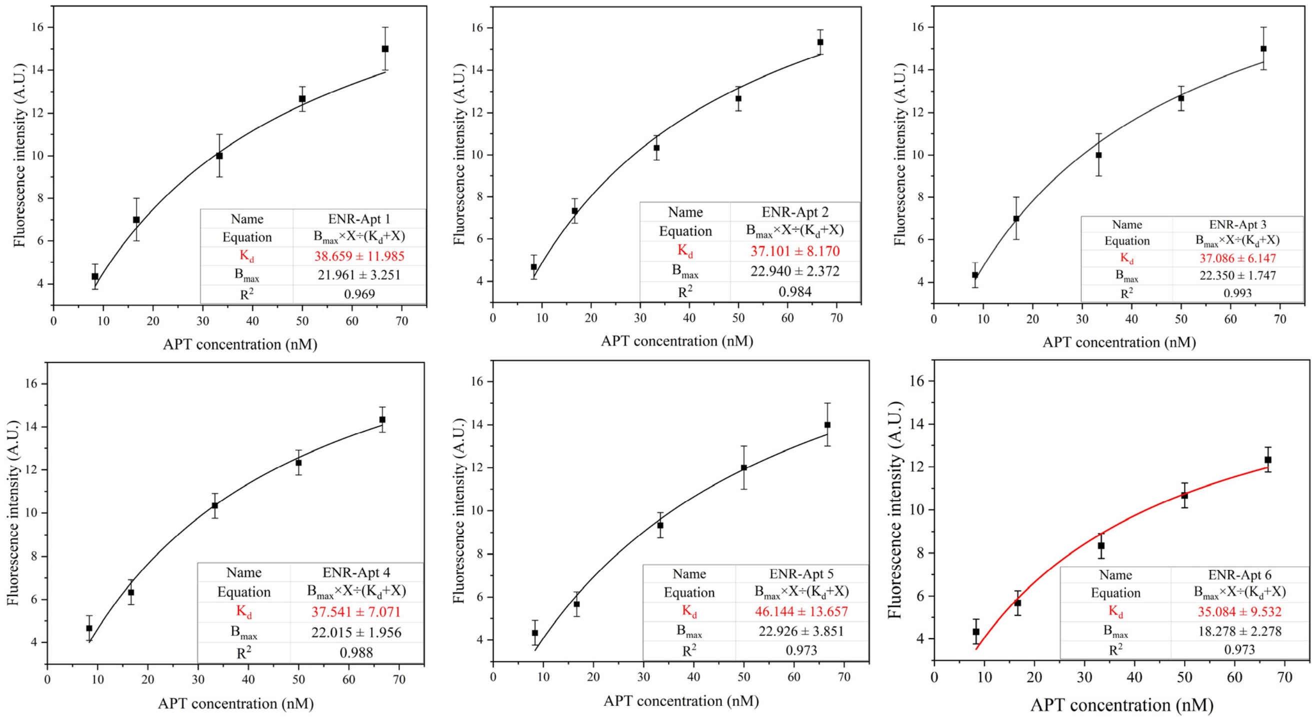

Figure.S4. Six candidate adaptors nonlinear fitting curves
